# Multiple Supernumerary Teeth with Concomitant Mandibular Hypo- Hyperdontia: A Case Report

**DOI:** 10.31729/jnma.8953

**Published:** 2025-04-30

**Authors:** Rabin Panthee, Manisha Upadhyay, Ankita Agrawal, Rachana Mishra, Priyanka Rana

**Affiliations:** 1 Department of Pedodontics and Preventive Dentistry, Universal College of Medical Sciences, College of Dental Surgery, Bhairahawa, Nepal

**Keywords:** *concomitant hypo-hyperdontia*, *hyperthyroidism*, *supernumerary tooth*, *tooth anomalies*

## Abstract

This is an extremely rare case of concomitant hypo-hyperdontia with mandibular mesiodens. Concomitant hypo-hyperdontia (CHH) refers to the presence of hypo and hyperdontia in the same patient. Supernumerary teeth may lead to difficulties such as delayed or ectopic eruption of permanent teeth, spacing, malocclusion, cystic lesions, and retained deciduous teeth. The present study describes an unusual case of an eleven-year-old girl with CHH and a medical history of hyperthyroidism. The Cone Beam Computed Topography (CBCT) results revealed thirty-one permanent teeth with a missing mandibular left lateral incisor, eleven deciduous teeth, and eight unerupted supernumerary teeth (four in the maxilla and four in the mandible). This study aims to outline the etiology, complications, diagnosis, and multidisciplinary approach for the management of a case of supernumerary teeth.

## INTRODUCTION

Supernumerary teeth, also known as hyperdontia, are teeth or tooth-like structures that have erupted or unerupted in addition to the normal set of dentition. Hypodontia is the agenesis of one or more teeth in primary and permanent dentition. Concomitant hypo-hyperdontia (CHH) is the presence of hypo and hyperdontia in the same patient, with a prevalence ranging from 0.002% to 3.1%.^1,2^ This study presents an unusual instance of CHH and mandibular mesiodens in an eleven-year-old female patient with a history of hyperthyroidism, including clinical and radiographic findings and a multidisciplinary approach for management.

## CASE REPORT

An eleven years old female visited the Department of Pedodontics and Preventive Dentistry with the chief complaint of a retained deciduous tooth in the upper front region of the jaw. The family history was not contributory. The patient's medical history revealed that she had been taking Carbamizole 25 mg once daily for hyperthyroidism for past four years.

**Figure 1:**
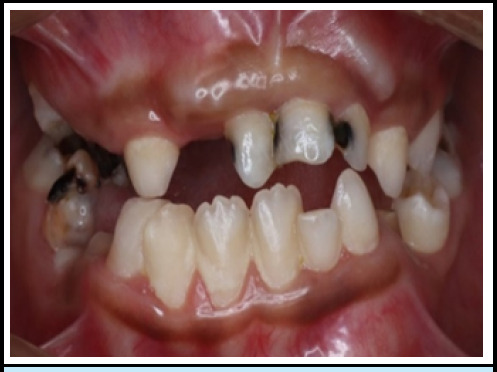
Intra-oral photograph showing maxillary and mandibular teeth and a visible bulge in maxillary anterior region

The patient weighed 32 kg, stood 128 cm tall, and had a mesomorphic body build. Diffuse swelling in the neck was present due to an enlarged thyroid gland. On intraoral examination: 15, 16, 24, 25, 26, 31, 35, 36, 41, 42, and 46 were completely erupted, and 17, 27, 37, 47, 34, and 43 were partially erupted. The deciduous teeth present in the oral cavity were 51, 53, 54, 61, 62, 63, 72, 73, 74, 83, and 85. Furthermore, a significant, visible bulge was found in the upper front region of the jaw (Figure 1). In this case, Iintraoral features also show several decayed teeth and root stumps.

**Figure 2:**
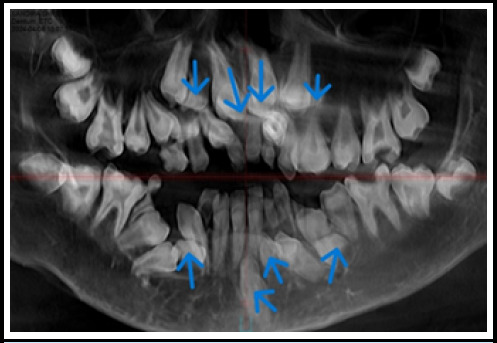
Panoramic view showing supernumerary teeth

**Figure 3:**
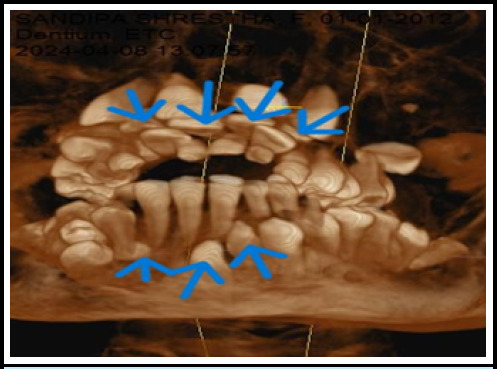
3D frontal view of maxilla and mandible showing supernumerary teeth

**Figure 4:**
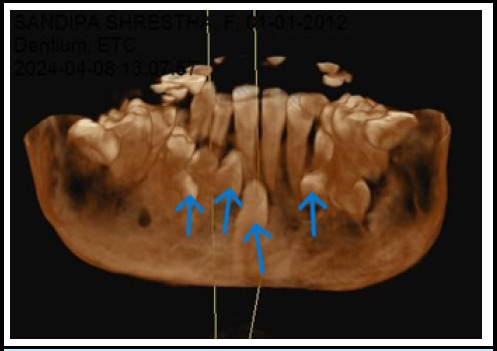
3D frontal view of maxilla and mandible showing supernumerary teeth

An orthopantomogram and lateral cephalogram revealed many supernumerary teeth (Figure 2). To validate the exact number and position of supernumerary teeth, Cone Beam Computed Tomography (CBCT) was performed. The CBCT results revealed thirty-one permanent teeth (sixteen in the maxilla and fifteen in the mandible) with a missing permanent mandibular left lateral incisor, eleven deciduous teeth, and eight impacted supernumerary teeth (four in the maxilla and four in the mandible). In the maxilla, four supplementary teeth were distributed bilaterally, impacted, and had a crown-like morphology (Figure 3). Similarly, in the mandible, there were four supernumerary teeth, three of which have the same morphology as maxillary supernumerary teeth, while one impacted supernumerary tooth is located in the midline and has a morphology similar to the incisor with a completely formed root, which is also known as mesiodens (Figure 4).

The chest X-ray revealed no abnormalities. Thyroid function tests reported T3 levels of 10.2 pg/ml, T4 levels of 1.68 ng/dl, and TSH levels of 0.039 microIU/ml. The blood examination was completely normal. Consultation with a general physician ruled out no any associated syndromes.

The current case required a multidisciplinary approach that included pedodontists, orthodontists, endodontists, oral surgeons, endocrinologists, pediatricians, and general practitioners. Initially, the following root stumps and retained deciduous teeth were extracted: 51, 54, 61, 62, 72, 74, and 85.

Followed by root canal treatment of a grossly carious tooth 16 and, composite restorations of 26, 36, and 46. The patient had been planned for orthodontic treatment as well as surgical removal of impacted supernumerary teeth.

## DISCUSSION

It is unusual to find CHH in the dental arch of normal individual. Supernumerary teeth occur at a rate of 0.3-1.7% in deciduous dentition and 1.5-3.5% in permanent dentition.^3^ Furthermore, they can be observed in syndromic patients, but they are unusual in people without any syndromes. Various studies indicate that 76-86% of non-syndromic patients have a single supernumerary tooth, while 12-23% have multiples. They are present in 0.2-3% of both primary and permanent dentitions, according to previous studies.^4^ In individuals exhibiting hypo-hyperdontia, hyperdontia is most common in the anterior region, with mesiodens being the most frequently associated with supernumerary teeth. Maxillary mesiodens (approximately 65%) are more commonly associated with hypo-hyperdontia than mandibular mesiodens (approximately 35%). There are just a few instances of mandibular mesiodens reported in the literature, approximately 0.01%.^2^ The shape can be tuberculate, conical, odontome, or similar to a natural tooth, depending on the morphology series. Hypo-hyperdontia can lead to missing second premolars, approximately 38%, as well as lateral incisors, approximately 28%.^1^ Supernumerary teeth are more common in males than females, with a reported sex ratio ranging from 2:1 to 6.5:1.^5^ The cause of supernumerary teeth is unknown. One theory suggests that it is brought about by a dichotomy in the tooth bud, while another suggests that it is caused by hyperactivity in the dental lamina. Autosomal inheritance also plays a role. They are commonly found in syndromes such as cleft lip and palate, trichorhinophalangeal syndrome, Marfan syndrome, Nance Horan syndrome, Cleidocranial dysplasia and Gardner's syndrome.^6^ CHH is classified into three types: maxillary arch alone, mandibular arch alone, and maxillary and mandibular arches combined.^1^ It may lead to complications such as delayed or ectopic eruption of permanent teeth, diastema, malocclusion, cystic lesions, resorption of adjoining teeth, intraoral infection, and retained deciduous teeth. Early diagnosis decreases complications and, when paired with early removal and orthodontic therapy, improves the prognosis.^7^

Various literature recommends two different extraction schedules for supernumerary teeth: early intervention and delayed intervention. Early intervention, which includes extraction shortly after assessment, is the best approach. Extraction of supernumerary teeth in early mixed dentition leads to spontaneous eruption and alignment of permanent successors. Additionally, it encourages early orthodontic intervention, leading to a better prognosis. Late intervention refers to extraction after adjacent teeth have fully formed roots. This is recommended to prevent problems with the developing roots of the permanent successor.^1^ Barham et al. (2022) suggested early tooth removal over the late period, resulting in no post-operative problems. Millineni et al. believe that after removing supernumerary teeth, the majority of affected permanent incisors erupt naturally, with some requiring orthodontic traction.^8^
